# Certificated Trained Nurses and the Poor Law

**Published:** 1917-03-10

**Authors:** 


### THE MATRONS' AND SISTERS' DEPARTMENT.

### CERTIFICATED TRAINED NURSES AND THE POOR LAW.

### The National Poor-Law Officers' Association's Campaign.

By the report of the annual meeting of the West Derby Union Branch of the National Poor-Law Officers' Association our attention is called once again to the question of the relationships of the "Poor-Law nurse," the National Poor-Law Officers' Association, and the Royal British College of Nursing, upon which question a paper was read at the meeting by Mr. C. A. W. Roberts, master of the Walton "Institution," which we understand to be the modern unofficial euphemism for "workhouse."

When the scheme for the foundation of a College of Nursing and for the settlement of the vexed question of the registration of nurses began to show promise of fulfilment, the National Poor-Law Officers' Association suddenly awoke to a realisation of the importance of nursing as a branch of the work of Poor-Law institutions. The Association had consistently failed to attract the nurses to its banner; it had, in fact, done nothing to deserve their support. It did not represent them. It had in the past practically ignored not only their claims, but their very existence. Then suddenly the Association decided to bestir itself with a view to enlisting the nurses in its ranks. The decision was due to the fact that the nurses had come to be an increasingly important and powerful section of the officers employed, under the Local Government Board, by Boards of Guardians. This ebullition of enthusiasm for the nurses' interests on the part of the Association was discussed in THE HOSPITAL last autumn, especially in the numbers of August 19 and September 9. The Association and Mr. Percival, its president, were at that time keenly desirous of "voicing the needs" of the Poor-Law nurses and of "organising" them in view of the pending constitution of a College of Nursing, which it was apparently taken for granted would be in some way inimical to their interests as a body. What these supposed needs were or what especial dangers lay ahead of them was not made clear. The suggestion appeared to be that there were a great many Poor-Law institution patients and, consequently, very large numbers of Poor-Law institution nurses, and that, as nurses engaged in this particular work had then taken but a small part in the College of Nursing movement, they would necessarily be, sooner or later, placed at one or other disadvantage. So the nurses were to join the Association, and the Association had the egregious vanity to imply that it could do the rest. We explained in our article of September 9 last the reasons why the Association is clearly not a suitable body to undertake the representation of nursing interests.

Mr. Roberts tells us again of the vague "dangers," and the value to the nurses of the machinery of the Association. He, however, denitely charges the College of Nursing with "almost entirely ignoring" the Poor-Law Nursing Service in the constitution of its Council. How, he asks, is this glaring defect, this "lob-sided complexion of the College of Nursing" (the phrase is Mr. Roberts' own), to be remedied? His remedy is for the nurses, one and all, to join the Association. Now, he says, has arisen "an excellent opportunity for the Association to prove that it is at last in a position to offer the nurses benefits in return for their membership, which *for some time past it has been extremely difficult to prove*" (the italics are ours). Mr. Roberts goes on to tell us that, despite the "untiring zeal" of Mr. Percival, the Association has had "little or no success," and that the one Poor-Law trained nurse on the Council of the College is "not even the nominee of the National Poor-Law Officers' Association"! This confession of utter failure on the part of the Association is solemnly put forward as an argument to show why the destinies of Poor-Law nursing should be left to its tender mercies. We also have found it, and still find it, "extremely difficult to prove" that nurses can hope for any "benefits" whatever from allegiance to that heterogeneous medley the Poor-Law Officers' Association. That the founders of the College of Nursing have not recognised, or been influenced by, the authority assumed by the Association is no matter for surprise. Mr. Roberts' claim that, but for the good offices of the Association and the Poor-Law Unions Association, Poor-Law trained nurses would have been debarred from registration we regard as preposterous.

Mr. Roberts and, presumably, the Association *now* strongly approve of the one-portal method of examination for the nursing profession, and even of the further "standardisation" of nurse-training. Mr. Roberts should read THE HOSPITAL and the Journal of his Association; he would then find that at the time when we were advocating central examinations for nurses the *Poor-Law Officers' Journal* was publishing verbose and maundering attempts to disparage in principle both central examinations and standardisation in nurse-training.

The one thing which Poor-Law institution-trained nurses have to fear is differentiation as a class. There has been a "Poor-Law taint"; this the National Poor-Law Officers' Association, whose members call their workhouses "institutions," will not deny. The taint is dying out. Nurses, who are perforce connected with a service confessedly effete and unpopular, who are in the Poor-Law service but not of it, can gain nothing by affiliating themselves to a body which can only emphasise the Poor-Law attributes of their work. We highly appraise the value of the Poor-Law nurses' work, but we realise that steps for the organisation of the nursing profession had inevitably to be initiated by the senior service, the great Hospital Nursing Service. Had the Poor-Law Service attempted to organise itself on such a model as the proposed College of Nursing it would have differentiated itself, to its own detriment, as a class of nursing apart and inferior. Whether at present fully represented or not upon the provisional Council of the College (and this question of representation we do not propose to here discuss), the Poor-Law trained nurses are being, and will be, fairly treated: the influence of the Local Government Board in the matter, which is by no means an unimportant factor, has been studiously ignored by the Poor-Law Officers' Association.

The matrons and nurses of the typical Poor-Law training-schools, the great separate infirmaries, have not sought the aid of the Association. On the contrary they are, with practical unanimity, opposed to its interference. We have no hesitation in stating once again our firm belief that the best interests of the nurses trained, undergoing training, or working in the Poor-Law Service cannot be furthered by the Poor-Law Officers' Association.

